# Supplementary material for: E3 Ubiquitin Ligase CHIP Inhibits Haemocyte Proliferation and Differentiation via the Ubiquitination of Runx in the Pacific Oyster
Source: Cells. 2024 Sep 13;13(18):1535. doi: 10.3390/cells13181535 (PMC11430624; doi:10.3390/cells13181535)
Supplement: Supplementary file 1 [file cells-13-01535-s001.zip › cells-3178214-supplementary/cells-3178214-supplementary-res.pdf]

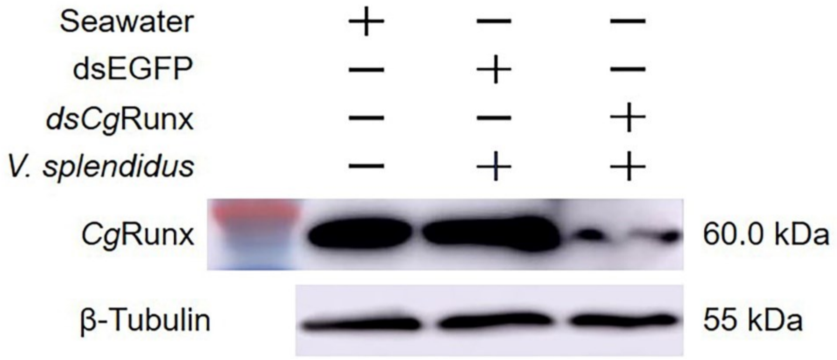

Supplemental Figure S1. The RNAi efficiency of *Cg*Runx assessed with haemocyte lysates by Western blotting. β-Tubulin was used as internal control.

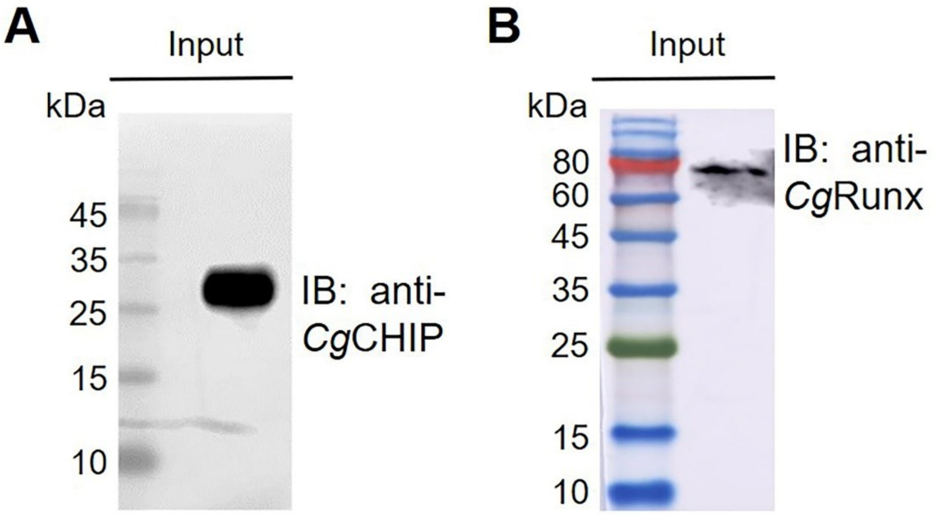

Supplemental Figure S2. The examination of haemocyte lysates with *Cg*CHIP and *Cg*Runx antibodies by Western blotting.
