# Supplementary material for: E3 Ubiquitin Ligase CHIP Inhibits Haemocyte Proliferation and Differentiation via the Ubiquitination of Runx in the Pacific Oyster
Source: Cells. 2024 Sep 13;13(18):1535. doi: 10.3390/cells13181535 (PMC11430624; doi:10.3390/cells13181535)
Supplement: Supplementary file 1 [file cells-13-01535-s001.zip › cells-3178214-supplementary/cells-3178214-suppplementary.pdf]

Table S1. Primer sequences used in this study.

Primers	Sequence (5'-3')
**Clone primers**	
P1 (*Cg*CHIP-F)	ATGAGTGCAACAGACTTAAAG
P2 (*Cg*CHIP-R)	CTAGTAGTCCTCTGCCCAAG
**Recombination primers**	
P3 (*Cg*CHIP-*Bam*H I-F)	CGGGATCCATGAGTGCAACAGACTTAAAG
P4 (*Cg*CHIP-*Hin*d III-R)	CCCAAGCTTGTAGTCCTCTGCCCAAG
**RNAi primers**	
P5 (dsCHIP-F)	TAATACGACTCACTATAGGGATGGCTTATTAGAAAGGATT
P6 (dsCHIP-R)	TAATACGACTCACTATAGGGTGAAGTGAGAGAGTGAGAAC
P7 (dsRunx-F)	TAATACGACTCACTATAGGGCAAGGACAACGCACAGTTAGACA
P8 (dsRunx-R)	TAATACGACTCACTATAGGGTGTATGTTCTCTCCTCGGGTAAG
P9 (dsEGFP-F)	GCGTAATACGACTCACTATAGGGACGTAAACGGCCACAAGT
P10 (dsEGFP-R)	GCGTAATACGACTCACTATAGGTTGTACAGCTCGTCCATGC
**qRT-PCR Primers**	
P11 (*Cg*CHIP-RT-F)	GTGTTACTTGAAACTAAAAAACTGGGACC
P12 (*Cg*CHIP-RT-R)	AAGACTGCTTACGGCTTCATCATACAA
P13 (*Cg*Runx-RT-F)	CACTGGCGTTCCAACAAGACATTAC
P14 (*Cg*Runx-RT-R)	GTCTACGGTCACTTTGATGGCTTTC
P15 (*Cg*EF-1α-F)	AGTCACCAAGGCTGCACAGAAAG
P16 (*Cg*EF-1α-R)	TCCGACGTATTTCTTTGCGATGT
